# Clinical significance of dynamic variation of low cholesterol and its prognostic value in patients with pyogenic liver abscesses: a retrospective study

**DOI:** 10.1186/s12879-023-08011-7

**Published:** 2023-02-06

**Authors:** Tiantong Feng, Wen Zhang, Xiaoxue Hou, Hui Yuan, Jinyuan Cai, Zhengyi Jiang, Pingping Hu, Ming Yue, Wenting Li, Chuanlong Zhu, Yuwen Li

**Affiliations:** 1 Department of Infectious Disease, The First Affiliated Hospital of Nanjing Medical University, 300 Guangzhou Rd., Nanjing, 210029 China; 2 Department of Infectious and Tropical Diseases, The Second Affiliated Hospital of Hainan Medical University, 368 Yehai Ave., Haikou, 570311 China; 3 Department of Pediatrics, The First Affiliated Hospital of Nanjing Medical University, 300 Guangzhou Rd., Nanjing, 210029 China

**Keywords:** Pyogenic liver abscess, Serum lipid, Total cholesterol, Drainage

## Abstract

**Background:**

Serum lipids variations are closely related to the sepsis progression; however, their value for patients with pyogenic liver abscesses (PLA) has rarely been studied. We investigated the serum lipid level variations in patients with PLA and its predictive value to the disease.

**Methods:**

The study included 328 patients with PLA hospitalized in the First Affiliated Hospital of Nanjing Medical University from January 2017 to December 2021; 35 (10.67%) in the severe group (SG) and 293 (89.33%) in the non-severe group (nSG). Their clinical records were analyzed retrospectively, and dynamic curves were drawn to clarify the changes in different indicators during the course of the disease.

**Results:**

High-density lipoprotein cholesterol (HDL-C), low-density lipoprotein cholesterol (LDL-C), and lipoprotein(a) (Lp(a)) in the SG were significantly lower than those in nSG (P < 0.001). Total cholesterol (TC) at baseline (OR = 0.184, P < 0.001) was an independent risk factor for severe patients and had the highest predictive value, with an area under the curve of 0.859 and a cut-off value of 2.70 mmol/L (sensitivity = 94.3%, specificity = 63.5%). For patients who met the criteria for drainage surgery, TC, HDL-C and LDL-C levels continued to decrease with antibiotic therapy alone before drainage and began to increase after the surgery.

**Conclusions:**

Low TC level on admission is an independent risk factor for the progression of severe illness in PLA patients, with the highest predictive value surpassing other routine clinical indices. Abscess drainage should be performed as soon as possible for patients whose TC continues to decline after medical treatment.

**Supplementary Information:**

The online version contains supplementary material available at 10.1186/s12879-023-08011-7.

## Background

The pyogenic liver abscess (PLA), a pyogenic infection, occurs owing to the liquefaction necrosis of liver parenchyma due to purulent bacterial invasion, including *Escherichia coli*, *Klebsiella pneumoniae* (*K. pneumoniae*), *Pseudomonas aeruginosa*, and *Staphylococcus aureus*. In general, it mainly occurs in middle-aged men with glucose intolerance or diabetes mellitus, possibly arising from the biliary tree; circulation (portal vein, hepatic artery); the adjacent focus of infection and penetrating trauma [1].

PLA gradually appeared in literature reports in the middle of the last century, and the mortality rate of PLA was as high as 50% owing to the limitation of medical treatment [2]. Currently, the development of imaging technology and antimicrobial and percutaneous drainage technologies have drastically improved the prognosis of patients with PLA [3]. However, adverse events may still occur owing to the lack of specific symptoms, being prone to sepsis or disseminated infection and the increase of multidrug-resistant *K. pneumoniae* infection, causing delayed diagnosis and treatment of PLA [4]. In recent reports, PLA has a mortality rate of 2–12% [5].

Serum lipids have been proven to be closely related to sepsis progression. Several studies have shown that patients with sepsis have decreased plasma total cholesterol (TC), high-density lipoprotein cholesterol (HDL-C) and low-density lipoprotein cholesterol (LDL-C), and subsequently, patients with low TC, HDL-C and LDL-C levels have higher death risk [6–8]. Additionally, patients with sepsis also have increased triglycerides (TG) and decreased lipoprotein(a) (Lp(a)) [9]. Previous studies have shown that patients with PLA generally present with leukocytosis, elevated alkaline phosphatase and abnormal liver function [10]; however, few studies have been conducted for assessing the condition and prognosis of PLA patients using lipid indices. Therefore, this study aimed to explore the variation of serum lipid levels in patients with PLA and its predictive value to the disease to provide a better reference for clinical diagnosis and treatment.

## Methods

This retrospective study was performed following the Declaration of Helsinki Ethical Principles and was approved by the Ethics Committee of the First Affiliated Hospital of Nanjing Medical University (2020-SR-048). The requirement for informed consent was waived as existing residual medical records were used.

### Study population

A total of 935 patients with PLA were admitted to the First Affiliated Hospital of Nanjing Medical University from January 2017 to December 2021. After screening by exclusion criteria, 328 cases were included.

The diagnostic criteria for PLA were as follows: (1) there were systemic symptoms such as fever and fatigue and/or local symptoms such as epigastric discomfort; (2) abdominal ultrasonography, computed tomography or magnetic resonance imaging findings suggesting liver abscess; (3) bacteriological culture results were positive and/or antimicrobial therapy was effective; and (4) percutaneous drainage or surgical treatment proved to be a bacterial suppurative infection. Patients who met criteria (3) or (4) based on criteria (1) and (2) were diagnosed as PLA.

Exclusion criteria were as follows: (1) PLA was not the leading cause of hospitalization; (2) the abscess was amoeba or tuberculous liver abscess; (3) patients with underlying hepatic disease, including viral hepatitis, autoimmune liver disease, fatty liver, cirrhosis, and liver cancer; (4) those with diseases known to cause dyslipidemia (besides diabetes), including hyperlipidemia, hypertension [11], chronic kidney disease [12], coronary heart disease, malignant tumor, hyperuricemia [13], and hyperthyroidism/hypothyroidism; (5) those taking medicines known to affect serum lipids, including lipid-regulating drugs, glucocorticoids, beta-blockers [14], and diuretics [15]; and (6) those who underwent surgery other than percutaneous drainage or laparoscopic abscess incision drainage during hospitalization.

Criteria for drainage were as follows: (1) Patient’s body temperature and symptoms did not return to normal or the condition deteriorated during antibiotic treatment; (2) re-examination of imaging suggested that the abscess had failed to shrink or continued to enlarge; and (3) the size and location of the abscess allowed puncture/incision drainage. Patients who met criteria (1) or (2) based on criteria (3) received drainage treatment in addition to antibiotic treatment.

Septic shock was diagnosed in septic patients with persisting hypotension requiring vasopressors to maintain MAP ≥ 65 mmHg and having a serum lactate level > 2 mmol/L despite adequate volume resuscitation [16]. The development of two or more concurrent organ dysfunctions was defined as multiple organ dysfunction syndromes (MODS).

**Fig. 1:**
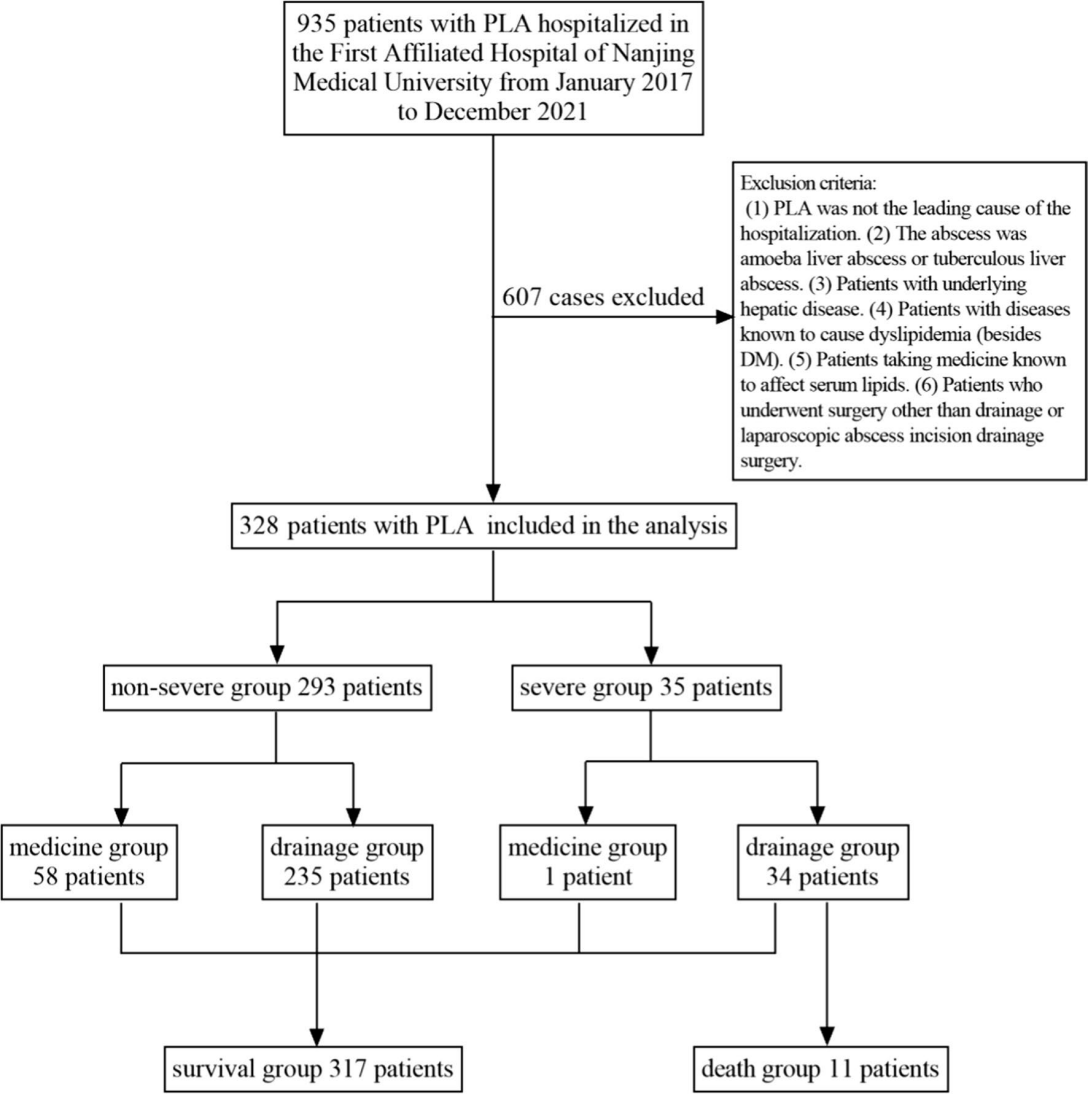
Study flow chart

Thirty-five of the 328 patients who developed septic shock or MODS (severe illness) were assigned to the severe group (SG), and the remaining 293 were assigned to the non-severe group (nSG). Eleven patients in the SG who died in a hospital or discontinued treatment owing to critical illness were classified as the death group. The remaining 317 patients who ameliorated after treatment were classified as the survival group. During the course of the disease, 59 and 269 patients who received antibiotic therapy alone without invasive remedies and received drainage treatment in addition to antibiotic treatment were classified as the medicine and drainage groups, respectively (Fig. 1).

The empirical treatment of PLA comprised carbapenem antibiotics like biapenem or meropenem in combination with metronidazole or ornidazole. Vancomycin or linezolid was added if necessary. Cefoperazone/sulbactam or moxifloxacin was used in patients with mild symptoms.

### Bacterial identification

The isolated bacteria were identified using the VITEK 2 Compact (bioMérieux, France). Twenty clinical antibiotics commonly used were analyzed using the MIC method according to the guidelines of the Clinical and Laboratory Standards Institute. Quality control strains included *Escherichia coli* ATCC 25922 and *Kleber pneumoniae* ATCC 700603.

### Data acquisition

The clinical data of patients with PLA were obtained from the medical record and laboratory report systems of our hospital. The baseline data were the results of the first venous sampling (mostly within 24 h after admission). Drainage group: Empiric antibiotic therapy was initiated for patients with PLA after admission; however, not all patients underwent abscess drainage immediately. Therefore, laboratory results were collected according to five periods for patients in the drainage group. When there were two or more laboratory results before drainage, the first result after admission was recorded as *On admission*, and the last result before drainage was recorded as *Before drainage*. However, when there was only one laboratory result before drainage if the time of blood sampling was more than 3 days before drainage (including the day of drainage treatment), it was recorded as *On admission*. If the time of blood sampling was ≤ 3 days before drainage, it was recorded as *Before drainage*. The duration from drainage to discharge was divided into three equal periods. The last result in each corresponding period was recorded as *Drainage: earlyphase*, *Drainage: metaphase*, and *Before discharge*. For patients in the medicine group, the first and second laboratory results after admission were collected and recorded as *1st sampling* and *2nd sampling*.

### Statistical analysis

SPSS 26.0 software was used for statistical analysis. Continuous variables conforming to normal distribution were presented as mean ± standard deviation and compared using a t-test. Non-normal variables were presented as the median (interquartile range) and compared using the Mann–Whitney U test. Categorical data were presented as numbers (percentages) and were compared using Chi-square or Fisher exact test. The receiver operating characteristic (ROC) curve analysis was performed for serum lipid and other statistically significant indicators, and corresponding cut-off values were calculated. The independent predictors associated with the severity of PLA were identified using binary logistic regression. P < 0.05 was considered statistically significant.

## Results

### Characteristics of PLA patients

A total of 328 patients with PLA were enrolled in this study, including 222 males (67.68%) and 106 females (32.32%), with a median age of 56 (49–66) years. Among them, 153 cases (46.65%) had diabetes. More than 90% of the patients had been treated with antibiotics before hospitalization since the onset of the disease. Bacteriological culture (including blood culture or/and pus culture) was conducted in 272 patients (82.93%) during hospitalization. One hundred and ninety-five strains of bacteria were isolated from 190 patients with positive culture results, including 155 strains of *K. pneumoniae* (79.49%), 19 strains of *Escherichia coli* (9.74%), and 21 strains of other bacteria (10.77%) (Table 1) (see Additional file 1: Table S1).

**Table 1:** Characteristics of patients with pyogenic liver abscesses

Characteristics	N = 328
Age (y)	56 (49–66)
Gender (n)	
Male	222 (67.68)
Female	106 (32.32)
Days after onset (d)	8.5 (6–14)
Antibiotics treatment prior to hospitalization (n)	297 (90.55)
Treatment	
Antibiotic treatment (n)	59 (17.99)
Antibiotic treatment & drainage treatment (n)	269 (82.01)
Drainage ways (N = 269)	
Percutaneous drainage (n)	253 (94.05)
Laparoscopic abscess incision drainage (n)	16 (5.95)
Time to start drainage treatment	
Pre-admission (n)	32 (11.90)
After admission (d)	3 (2–5)
Hospital stays (d)	12.5 (8–19)
Disease condition	
Non-severe group (n)	293 (89.33)
Severe group (survival) (n)	24 (7.32)
Severe group (death) (n)	11 (3.35)
Location of abscesses (n)	318
Right hemiliver (n)	233 (71.04)
Left hemiliver (n)	68 (20.73)
Both hemilivers (n)	27 (8.23)
Maximal body temperature (℃)	39.2 (38.6–40.0)
Comorbidities	
Diabetes mellitus (n)	153 (46.65)
Cholecystolithiasis (n)	34 (10.37)
After hepatobiliary and pancreatic surgery (n)	41 (12.50)
Symptoms	
Fever (n)	317 (96.65)
Chill (n)	155 (47.26)
Gastrointestinal symptoms (n)	178 (54.27)
Epigastric discomfort (n)	147 (44.82)
General malaise (n)	102 (31.10)
Respiratory symptom (n)	34 (10.37)
Jaundice (n)	10 (3.05)
Bacteriological culture (blood or/and pus) (N = 272)	
Positive (n)	190 (69.85)
Negative (n)	82 (30.15)
Bacterial species (N = 195)	
*Klebsiella pneumoniae* (n)	155 (79.49)
* Escherichia coli* (n)	19 (9.74)
Others (n)	21 (10.77)

### Comparison of baseline data

No significant differences in age, sex and body mass index (BMI) were observed between the SG and nSG (*P* > 0.05). The proportion of patients with diabetes and heat peak in the SG were higher than those in the nSG (*P* < 0.05). Differences in laboratory indicators between the two groups were ubiquitous. Compared to nSG, TC, HDL-C, LDL-C and Lp(a) in SG were significantly decreased (*P* < 0.001), while TG was significantly increased (*P* = 0.003). The discrepancies in other indicators such as liver function, blood routine and coagulation function were consistent with previous studies. No statistical difference was found in albumin and alkaline phosphatase (ALP) between the two groups (*P* > 0.05) (Table 2). TC, HDL-C, LDL-C and Lp (a) were also significantly lower in the death group than that in the survival group (*P* < 0.001) (see Additional file 1: Table S2).

**Table 2:** Comparison of baseline data between patients with PLA in the severe and non-severe groups

	Non-severe group (n = 293)	Severe group (n = 35)	*P* value
Age (y)	56.0 (49.0–66.0)	62.0 (45.0–66.0)	0.327
Gender (n)			0.616
Male	197 (67.24)	25 (71.43)	
Female	96 (32.76)	10 (28.57)	
Diabetes mellitus (n)	131 (44.71)	22 (62.86)	0.042
BMI (kg/m^2^)	24.12 ± 3.14	22.94 ± 2.76	0.170
Maximal body temperature (℃)	39.2 (38.6–40.0)	39.4 (39.0–40.0)	0.041
Serum lipid			
TC (mmol/L)	2.90 (2.41–3.64)	1.83 (1.58–2.42)	< 0.001
HDL-C (mmol/L)	0.59 (0.45–0.73)	0.36 (0.24–0.45)	< 0.001
LDL-C (mmol/L)	1.92 (1.55–2.38)	1.22 (0.97–1.50)	< 0.001
TG (mmol/L)	1.05 (0.83–1.43)	1.40 (1.00–2.22)	0.003
Lp(a) (mg/L)	115.0 (56.5–239.5)	23.0 (12.0–67.0)	< 0.001
Hepatic function			
Albumin (g/L)	29.79 ± 4.86	28.07 ± 5.37	0.051
TBIL (μmol/L)	11.80 (8.45–18.15)	27.70 (12.0–53.60)	< 0.001
DBIL (μmol/L)	5.90 (3.90–10.10)	13.80 (7.50–41.70)	< 0.001
ALT (U/L)	46.70 (26.15–69.85)	78.30 (38.80–253.40)	< 0.001
AST (U/L)	35.60 (23.65–52.85)	83.50 (35.60–276.60)	< 0.001
ALP (U/L)	174.0 (119.6–259.0)	168.2 (109.1–256.0)	0.841
LDH (U/L)	250.0(207.5–321.0)	333.0 (280.0–426.0)	< 0.001
Blood routine			
WBC (10^9^/L)	10.60 (8.18–13.50)	14.20 (9.57–23.80)	0.001
NE% (%)	82.55 (76.19–87.00)	89.80 (85.30–91.50)	< 0.001
HGB (g/L)	113.50 ± 17.83	99.80 ± 17.49	< 0.001
PLT (10^9^/L)	224.0 (145.0–319.0)	65.0 (34.0–177.0)	< 0.001
Coagulation function			
PT (s)	13.60 (12.85–14.40)	15.80 (13.90–21.50)	< 0.001
APTT (s)	29.90 (27.65–32.70)	38.00 (33.20–44.00)	< 0.001
FIB (g/L)	6.06 (4.75–6.95)	4.46 (2.44–6.44)	< 0.001

BMI: body mass index; TC: total cholesterol; HDL-C: high-density lipoprotein cholesterol; LDL-C: low-density lipoprotein cholesterol; TG: triglyceride; Lp(a): lipoprotein(a); TBIL: total bilirubin; DBIL: direct bilirubin; ALT: alanine transaminase; AST: aspartate transaminase; ALP: alkaline phosphatase; LDH: lactate dehydrogenase; WBC: white blood cell count; NE%: neutrophil percentage; HGB: hemoglobin; PLT: platelet; PT: prothrombin time; APTT: activated partial thromboplastin time; FIB: fibrinogen

### ROC analysis of serum lipid and other indicators

**Fig. 2:**
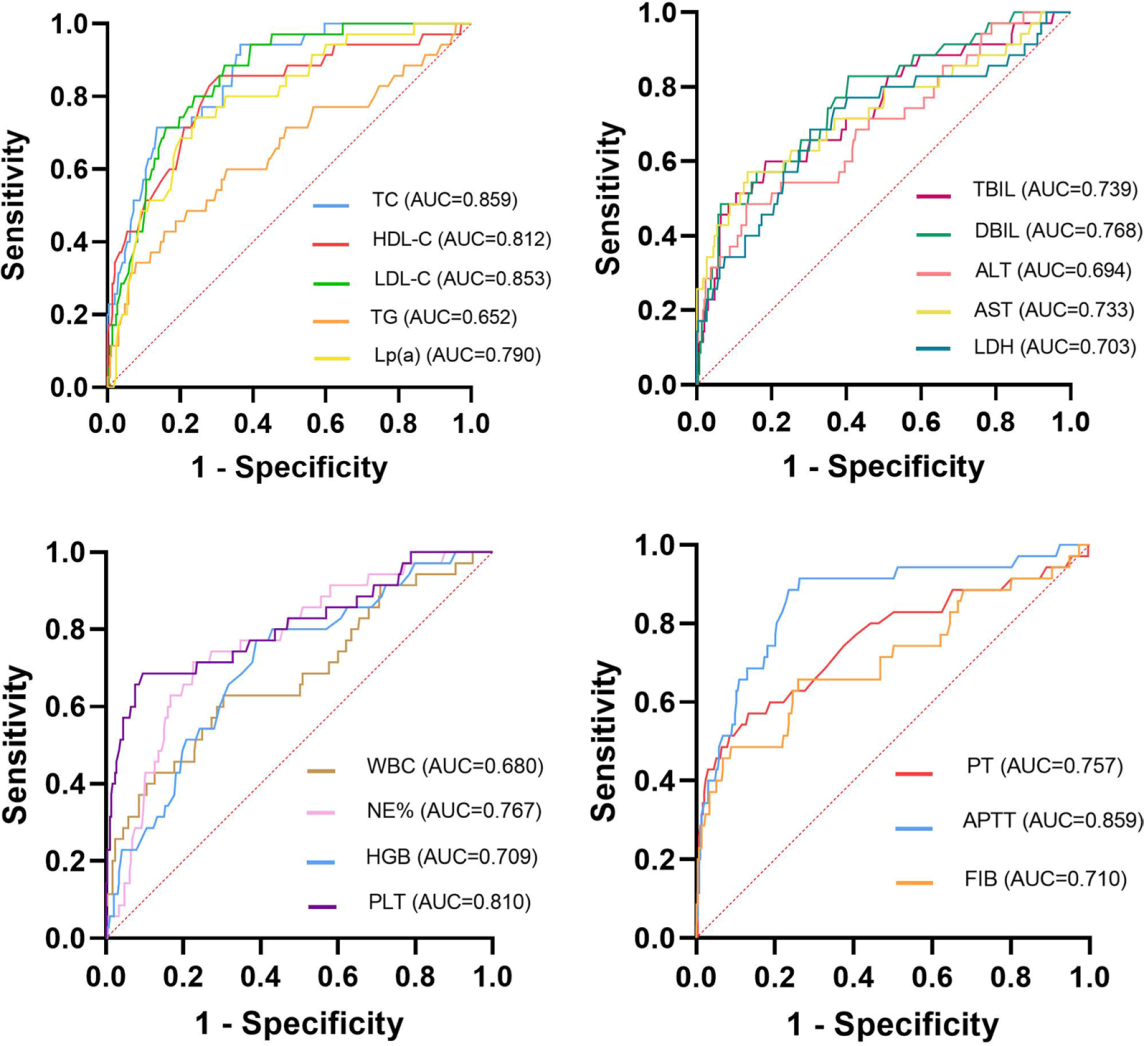
ROC curves for the prediction of severe illness by laboratory indicators routinely measured in patients with PLA. TC: total cholesterol; HDL-C: high-density lipoprotein cholesterol; LDL-C: low-density lipoprotein cholesterol; TG: triglyceride; Lp(a): lipoprotein(a); TBIL: total bilirubin; DBIL: direct bilirubin; ALT: alanine transaminase; AST: aspartate transaminase; LDH: lactate dehydrogenase; WBC: white blood cell count; NE%: neutrophil percentage; HGB: hemoglobin; PLT: platelet; PT: prothrombin time; APTT: activated partial thromboplastin time; FIB: fibrinogen

ROC curve analysis was performed for serum lipids and other laboratory indices with statistical differences. TC and activated partial thromboplastin time (APTT) was demonstrated with the highest predictive value for severe patients, followed by LDL-C and HDL-C. The area under the curve (AUC) was 0.859, 0.859, 0.853 and 0.812, respectively. Using the cut-off value of 2.70 mmol/L, TC predicted severe illness with a sensitivity of 94.3% and specificity of 63.5%. TG has the lowest predictive value, with an AUC of 0.652. The AUC of other indicators mostly ranged from 0.7 to 0.8 (Fig. 2; see Additional file 1: Table S3). In addition to TG, serum lipid indices had higher predictive value for death patients. The AUC of TC reached 0.906. The sensitivity and specificity reached 81.8% and 87.4% respectively when the cut-off value was 1.98 (see Additional file 1: Table S4, Fig. S1).

### Logistic regression analysis of severe illness in PLA patients

TC with the highest AUC in serum lipid indices was selected for analysis. Other factors with P < 0.1 were screened using stepwise forward regression. Finally, TC, white blood cell count (WBC), platelet (PLT) and APTT were included in the regression analysis as independent variables. The results showed that independent risk factors for severe illness of patients with PLA were decreased TC (OR: 0.184, *P* < 0.001), PLT (OR: 0.991, *P* = 0.002), and increased WBC (OR: 1.119, *P* = 0.008), APTT (OR: 1.115, *P* = 0.010) (Table 3).

**Table 3:** Binary logistic regression analysis of factors for severe illness in patients with PLA

Variable	B	OR	95%CI	*P* value
TC	− 1.695	0.184	0.075–0.450	< 0.001
WBC	0.113	1.119	1.030–1.217	0.008
PLT	− 0.009	0.991	0.986–0.997	0.002
APTT	0.109	1.115	1.026–1.211	0.010

TC: total cholesterol; WBC: white blood cell count; PLT: platelet; APTT: activated partial thromboplastin time

### Dynamic analysis of laboratory results in PLA patients

**Fig. 3:**
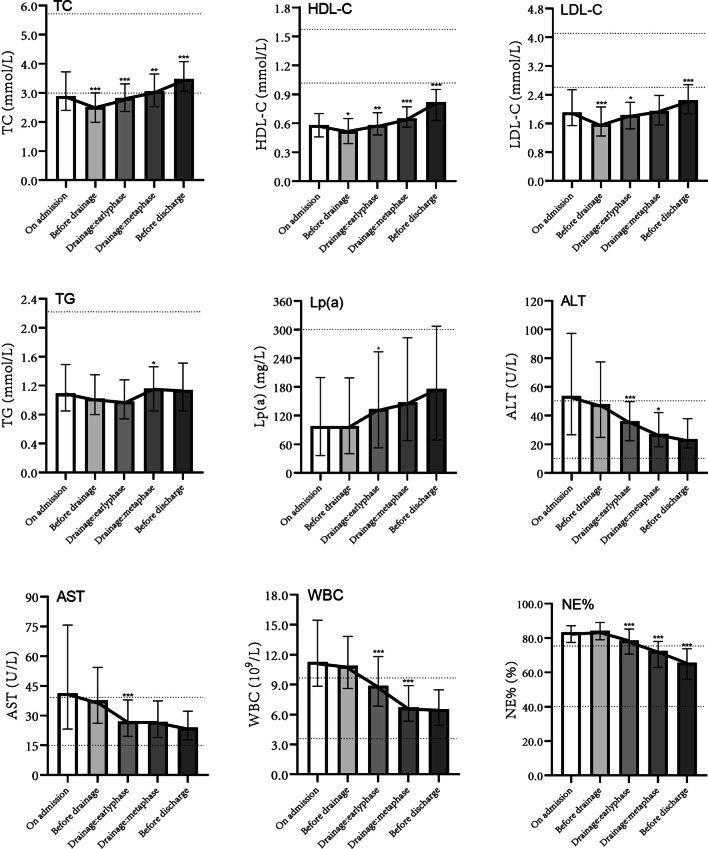
Variation curves of laboratory indices of patients with PLA in the drainage group (survival) during hospitalization. TC: total cholesterol; HDL-C: high-density lipoprotein cholesterol; LDL-C: low-density lipoprotein cholesterol; TG: triglyceride; Lp(a): lipoprotein(a); ALT: alanine transaminase; AST: aspartate transaminase; WBC: white blood cell count; NE%: neutrophil percentage. *Data in this period showed statistically significant difference compared with the previous period, P < 0.05; **P < 0.01; ***P < 0.001

Drainage group (survival): the variation curves were drawn using the laboratory results of five periods before (*On admission, Before drainage*) and after (*Drainage: earlyphase, Drainage: metaphase, Before discharge*) drainage. During the period of antibacterial therapy alone before drainage, TC, HDL-C and LDL-C of patients with PLA reduced significantly, which began to rise gradually after drainage. TC before discharge was significantly higher than that on admission (*P* < 0.001), with a median increase of 0.6 mmol/L. Lp(A) had no significant change before drainage, but increased after it. No regularity was found in TG. After admission, alanine transaminase (ALT) and aspartate transaminase (AST) showed a decreasing trend. WBC and neutrophil percentage (NE%) were relatively stable before drainage; however, they decreased significantly after drainage (Fig. 3).

**Fig. 4:**
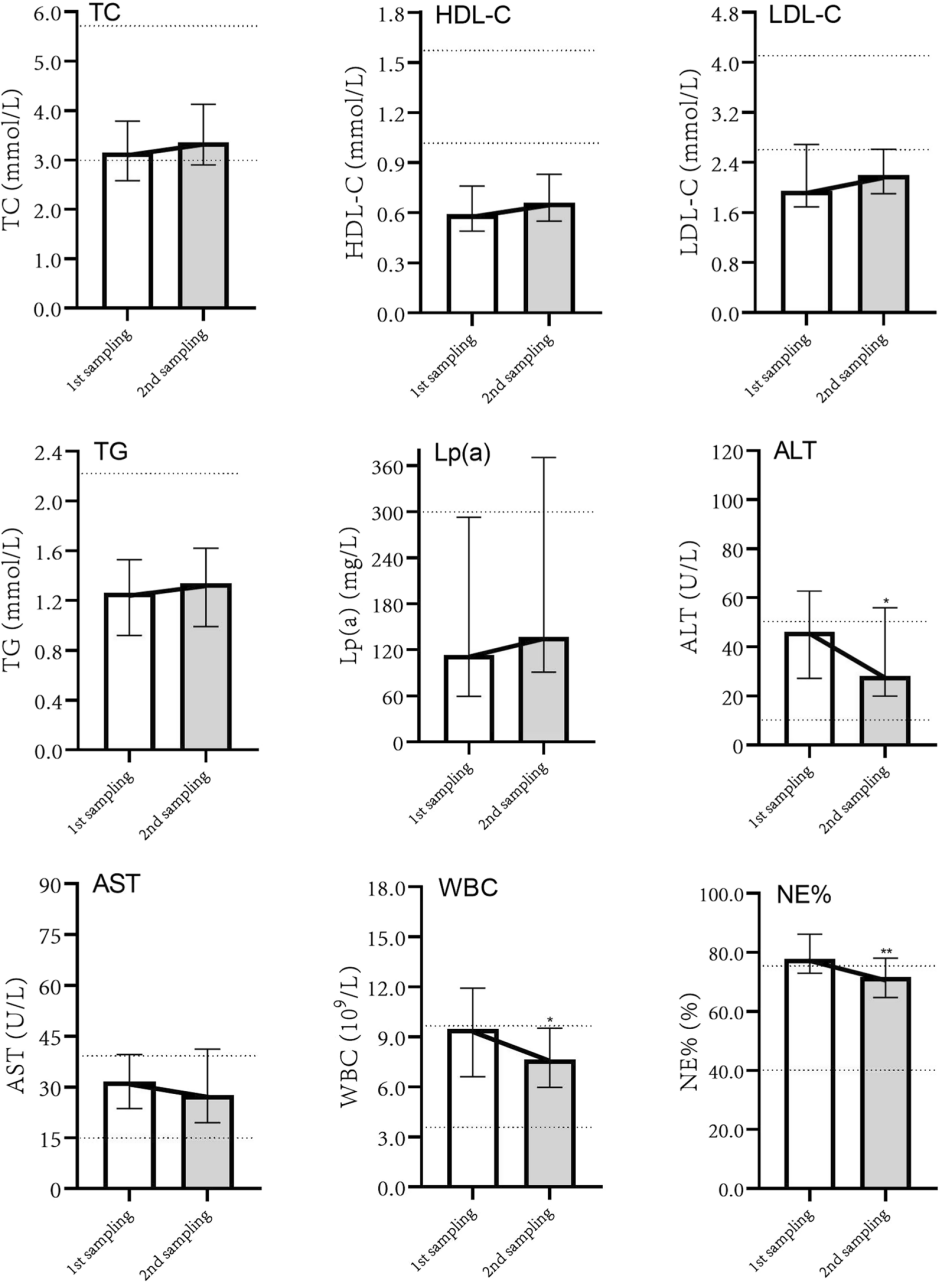
Variation curves of laboratory indices of patients with PLA in the medicine group during hospitalization. TC: total cholesterol; HDL-C: high-density lipoprotein cholesterol; LDL-C: low-density lipoprotein cholesterol; TG: triglyceride; Lp(a): lipoprotein(a); ALT: alanine transaminase; AST: aspartate transaminase; WBC: white blood cell count; NE%: neutrophil percentage. *Data in this period showed statistically significant difference compared with the previous period, *P* < 0.05; **P < 0.01; ***P < 0.001

Medicine group: The first (*1st sampling*) and second (*2nd sampling*) laboratory results after admission were used for plotting variation curves. Different from the drainage group, patients in the medicine group showed an increase in TC, HDL-C, LDL-C and Lp(a), combined with a decrease in ALT, AST, WBC and NE%, which could be observed shortly after antibacterial treatment (Fig. 4).

## Discussion

TC, HDL-C, LDL-C, TG and Lp(a) are the five serum lipid indices routinely detected in the biochemical examination of our hospital. Among them, TC, HDL-C and LDL-C (THL) show prosperous predictive value for the pathogenetic condition of patients with PLA. Results showed that THL decreased significantly in patients with severe illness. TC at baseline had the highest predictive value and was an independent risk factor for progression to severe illness.

HDL particles can bind and remove bacterial toxins, inhibit macrophage inflammatory responses, and prevent endothelial activation [17]. LDL particles have anti-inflammatory properties likewise [18]. As a result, THL decrease in patients with sepsis. As a center of lipid metabolism, THL decline has also been observed in patients with liver disease [19, 20]. Therefore, we believe that the co-existing pathophysiology of inflammatory consumption, liver injury and intake reduction in PLA play a synergistic role in the decline of THL, giving these indicators greater predictive value. As for other common abnormal laboratory indicators of patients with PLA, such as increased bilirubin, alkaline phosphatase and leukocytosis, all showed statistical differences in the baseline data analysis; however, with an inferior predictive value for TC and LDL-C.

As observed from the curve, the THL of patients in the drainage group decreased significantly when medication was administered before drainage. However, in the medicine group, THL recovery was observed after antibiotic treatment. Considering that reduced TC is an independent risk factor for patients with PLA to develop severe illness, THL can be used as references to judge the effectiveness of medical treatment and the necessity of abscess drainage. Abscess drainage should be performed as soon as possible if the patient’s THL continue to decline after empiric antimicrobial treatment, especially if TC drops below 2.70 mmol/L (sensitivity to predict severe illness was 94.3%). THL of patients displayed a rise after abscess drainage, and achieved the normal reference range before discharge, significantly higher than the level on admission, suggesting that THL can also be used as references for discharge. When TC level is more than 0.6 mmol/L higher than that on admission, the patient is considered to be recovering well and ready to be discharged.

The opposite variation trend of THL in the drainage and medicine groups after medicine therapy can widen the gaps of laboratory results between different patients to better identify the severity of patients. By contrast, ALT and AST in the drainage group showed an overall downward trend after admission. Most patients were treated with antibiotics before admission, and some with hepatoprotective drugs, which could explain the relatively low liver enzymes at admission. WBC and NE% were relatively stable before drainage; however, they were also susceptible to antimicrobial treatment, and the OR of WBC was close to 1. Therefore, their dynamic changes cannot be used as a strong reference for conditional judgment. We hypothesized that in patients requiring drainage therapy, sensitive antimicrobial therapy effectively inhibits abscess enlargement and further inflammatory damage to the surrounding liver tissue, which inhibits the increase of liver enzymes and WBC; however, it does not lead to rapid absorption and shrinkage of the abscess. The persistence of abscess leads to the intermittent entry of bacteria, toxins, and necrotic material into the bloodstream. Systemic inflammatory responses lead to continuous depletion of THL levels and consumption of the body; therefore, THL are of greater value in prognostic prediction.

TG and Lp(A) have little significance for patients with PLA. Although Lp(a) decreases in severe PLA patients and rises after drainage, it varies greatly among individuals. Results can fluctuate from tens to hundreds even in healthy individuals. Liver disease and reduced intake can lead to a decrease in TG, while various cytokines in sepsis can cause an increase in TG levels [21]. These two opposite effects achieved a relative balance in patients with PLA, resulting in irregular TG in data comparison and dynamic curve presentation.

This was a single-center retrospective study with some limitations. Most of the patients had received treatment before hospitalization, and the post-hospitalization examination results could not reflect the early onset of patients with PLA. Benefiting from the improvement of diagnosis and treatment, only a few patients with PLA developed into severe cases, and death was even rarer, which inevitably resulted in a large difference in sample sizes between groups.

## Conclusion

Low TC levels on admission are an independent risk factor for the progression of severe illness in PLA patients, with the highest predictive value surpassing other routine clinical indices. Abscess drainage should be performed promptly for patients whose TC continues to decline after medical treatment.

## Supplementary Information

**Additional file 1: Table S1.** The composition of isolated bacteria. **Table S2.** Comparison of baseline data between patients with PLA in the death and survival groups. **Table S3.** Performance of different prognostic indicators on predicting severe illness of patients with PLA. **Table S4.** Performance of different prognostic indicators on predicting death of patients with PLA. **Figure S1.** Receiver operating characteristic (ROC) curves for the prediction of death by laboratory indicators routinely measured in patients with PLA.

## Data Availability

The data set generated and analyzed in the current study involved patient medical information. Public release of information was not approved by the ethics committee. Requests for further information should be directed to the corresponding author.

## Abbreviations

PLA: Pyogenic liver abscess
*K. pneumoniae*: *Klebsiella pneumoniae*
TC: Total cholesterol
HDL-C: High-density lipoprotein cholesterol
LDL-C: Low-density lipoprotein cholesterol
TG: Triglyceride
Lp(a): Lipoprotein(a)
MODS: Multiple organ dysfunction syndrome
SG: Severe group
nSG: Non-severe group
ROC: Receiver operating characteristic
BMI: Body mass index
TBIL: Total bilirubin
DBIL: Direct bilirubin
ALT: Alanine transaminase
AST: Aspartate transaminase
ALP: Alkaline phosphatase
LDH: Lactate dehydrogenase
WBC: White blood cell count
NE%: Neutrophil percentage
HGB: Hemoglobin
PLT: Platelet
PT: Prothrombin time
APTT: Activated partial thromboplastin time
FIB: Fibrinogen
AUC: Areas under the curve
THL: TC, HDL-C and LDL-C
