# Supplementary material for: Clinical significance of dynamic variation of low cholesterol and its prognostic value in patients with pyogenic liver abscesses: a retrospective study
Source: BMC Infect Dis. 2023 Feb 6;23:70. doi: 10.1186/s12879-023-08011-7 (PMC9901086; doi:10.1186/s12879-023-08011-7)
Supplement: Supplementary file 1 — Additional file 1: Table S1. The composition of isolated bacteria. Table S2. Comparison of baseline data between patients with PLA in the death and survival groups. Table S3. Performance of different prognostic indicators on predicting severe illness of patients with PLA. Table S4. Performance of different prognostic indicators on predicting death of patients with PLA. Figure S1. Receiver operating characteristic (ROC) curves for the prediction of death by laboratory indicators routinely measured in patients with PLA. [file 12879_2023_8011_MOESM1_ESM.docx]

Supplementary

Table S1. The composition of isolated bacteria.

| Bacterial species | Total number of strains  (n=195) | From pus  (n=178) | From blood  (n=56) |
| --- | --- | --- | --- |
| Klebsiella pneumoniae | 155（79.49%） | 141 (79.21%) | 40 (71.43%) |
| Escherichia coli | 19（9.74%） | 18 (10.11%) | 9 (16.07%) |
| Enterobacter cloacae | 1（0.51%） | 1 (0.56%) | 0 |
| Citrobacter koseri | 1（0.51%） | 1 (0.56%) | 1 (1.79%) |
| Enterococcus faecium | 2（1.03%） | 2 (1.12%) | 1 (1.79%) |
| Enterococcus faecalis | 2（1.03%） | 2 (1.12%) | 0 |
| Enterococcus avium | 2（1.03%） | 2 (1.12%) | 0 |
| Streptococcus hemolyticus | 1（0.51%） | 1 (0.56%) | 0 |
| Streptococcus pneumoniae | 1（0.51%） | 1 (0.56%) | 0 |
| Streptococcus constellatus | 4（2.05%） | 4 (2.25%) | 2 (3.57%) |
| Streptococcus intermadius | 1（0.51%） | 1 (0.56%) | 0 |
| Streptococcus gordonii | 1（0.51%） | 0 | 1 (1.79%) |
| Streptococcus anginosus | 1（0.51%） | 1 (0.56%) | 0 |
| Staphylococcus aureus | 2（1.03%） | 1 (0.56%) | 1 (1.79%) |
| Pseudomonas aeruginosa | 1（0.51%） | 1 (0.56%) | 0 |
| Morganella morganii ssp morganii | 1（0.51%） | 1 (0.56%) | 1 (1.79%) |

Table S2. Comparison of baseline data between patients with PLA in the death and survival groups.

|  | Survival group  (n=317) | Death group  (n=11) | *P* value |
| --- | --- | --- | --- |
| Age (y) | 56.0(48.5-66.0) | 65.0(55-75.0) | 0.045 |
| Gender (n) |  |  | 0.971 |
| Male | 214(67.51) | 8(72.73) |  |
| Female | 103(32.49) | 3(27.27) |  |
| Diabetes mellitus (n) | 145(45.74%) | 8(72.73%) | 0.078 |
| BMI (kg/m^2^) | 24.07±3.12 | 22.57±2.80 | 0.187 |
| Maximal body temperature (℃) | 39.2(38.6-40.0) | 39.5(39.0-40.0) | 0.182 |
| Serum lipid |  |  |  |
| TC (mmol/L) | 2.83(2.32-3.58) | 1.26(1.09-1.96) | <0.001 |
| HDL-C (mmol/L) | 0.57(0.43-0.71) | 0.24(0.17-0.46) | <0.001 |
| LDL-C (mmol/L) | 1.88(1.50-2.35) | 0.99(0.79-1.36) | <0.001 |
| TG (mmol/L) | 1.10(0.84-1.46) | 1.01(0.61-2.55) | 0.896 |
| Lp(a) (mg/L) | 108.0(46.0-225.0) | 19.0(9.0-23.0) | <0.001 |
| Hepatic function |  |  |  |
| Albumin (g/L) | 29.63±4.92 | 28.94±5.55 | 0.650 |
| TBIL (μmol/L) | 12.10(8.55-19.35) | 53.00(14.00-92.30) | 0.001 |
| DBIL (μmol/L) | 6.10(4.00-10.50) | 30.00(8.20-63.30) | <0.001 |
| ALT (U/L) | 48.30(27.20-74.45) | 74.80(33.40-248.50) | 0.082 |
| AST (U/L) | 36.90(24.15-55.35) | 120.80(28.40-309.00) | 0.035 |
| ALP (U/L) | 172.0(118.5-257.5) | 232.0(100.7-317.0) | 0.577 |
| LDH (U/L) | 225.0(210.5-330.0) | 327.0(188.0-615.0) | 0.057 |
| Blood routine |  |  |  |
| WBC (10^9^/L) | 10.71(8.34-14.20) | 18.11(9.78-25.99) | 0.019 |
| NE% (%) | 82.90(76.70-88.00) | 90.00(86.70-91.80) | 0.002 |
| HGB (g/L) | 112.74±18.00 | 91.73±14.24 | <0.001 |
| PLT (10^9^/L) | 218.0(131.0-310.5) | 63.0(18.0-280.0) | 0.007 |
| Coagulation function |  |  |  |
| PT (s) | 13.60(12.90-14.55) | 18.40(14.20-22.50) | <0.001 |
| APTT (s) | 30.10(27.95-33.00) | 43.60(35.10-46.50) | <0.001 |
| FIB (g/L) | 6.00(4.54-6.93) | 3.43(2.19-5.90) | 0.002 |

Abbreviations: BMI, body mass index; TC, total cholesterol; HDL-C, high-density lipoprotein cholesterol; LDL-C, low-density lipoprotein cholesterol; TG, triglyceride; Lp(a), lipoprotein(a); TBIL, total bilirubin; DBIL, direct bilirubin; ALT, alanine transaminase; AST, aspartate transaminase; ALP, alkaline phosphatase; LDH, lactate dehydrogenase; WBC, white blood cell count; NE%, neutrophil percentage; HGB, hemoglobin; PLT, platelet; PT, prothrombin time; APTT, activated partial thromboplastin time; FIB, fibrinogen.

Table S3. Performance of different prognostic indicators on predicting severe illness of patients with PLA.

| Variables | cut-off value | AUC | 95%CI | Sensitivity | Specificity |
| --- | --- | --- | --- | --- | --- |
| TG | 2.70 | 0.859 | 0.801-0.916 | 0.943 | 0.635 |
| HDL-C | 0.49 | 0.812 | 0.729-0.895 | 0.857 | 0.693 |
| LDL-C | 1.67 | 0.853 | 0.797-0.908 | 0.886 | 0.678 |
| TG | 1.30 | 0.652 | 0.544-0.761 | 0.600 | 0.672 |
| Lp(a) | 54.5 | 0.790 | 0.714-0.866 | 0.743 | 0.758 |
| TBIL | 20.85 | 0.739 | 0.644-0.835 | 0.600 | 0.816 |
| DBIL | 7.00 | 0.768 | 0.682-0.853 | 0.829 | 0.594 |
| ALT | 99.20 | 0.694 | 0.595-0.794 | 0.486 | 0.867 |
| AST | 72.05 | 0.733 | 0.629-0.837 | 0.571 | 0.863 |
| LDH | 297.5 | 0.703 | 0.600-0.807 | 0.686 | 0.696 |
| WBC | 12.21 | 0.680 | 0.577-0.783 | 0.629 | 0.696 |
| NE% | 87.55 | 0.767 | 0.686-0.848 | 0.714 | 0.775 |
| HGB | 109.50 | 0.709 | 0.621-0.798 | 0.771 | 0.611 |
| PLT | 99.5 | 0.810 | 0.720-0.900 | 0.686 | 0.904 |
| PT | 15.35 | 0.757 | 0.656-0.859 | 0.571 | 0.867 |
| APTT | 32.40 | 0.859 | 0.787-0.931 | 0.914 | 0.737 |
| FIB | 4.84 | 0.710 | 0.602-0.817 | 0.657 | 0.741 |

Abbreviations: TC, total cholesterol; HDL-C, high-density lipoprotein cholesterol; LDL-C, low-density lipoprotein cholesterol; TG, triglyceride; Lp(a), lipoprotein(a); TBIL, total bilirubin; DBIL, direct bilirubin; ALT, alanine transaminase; AST, aspartate transaminase; LDH, lactate dehydrogenase; WBC, white blood cell count; NE%, neutrophil percentage; HGB, hemoglobin; PLT, platelet; PT, prothrombin time; APTT, activated partial thromboplastin time; FIB, fibrinogen.

Table S4. Performance of different prognostic indicators on predicting death of patients with PLA.

| Variables | cut-off value | AUC | 95%CI | Sensitivity | Specificity |
| --- | --- | --- | --- | --- | --- |
| TC | 1.98 | 0.906 | 0.820-0.992 | 0.818 | 0.874 |
| HDL-C | 0.33 | 0.823 | 0.651-0.995 | 0.727 | 0.927 |
| LDL-C | 1.48 | 0.895 | 0.817-0.973 | 0.909 | 0.766 |
| TG | 1.72 | 0.512 | 0.276-0.747 | 0.455 | 0.836 |
| Lp(a) | 23.5 | 0.876 | 0.807-0.946 | 0.818 | 0.864 |
| TBIL | 33.05 | 0.804 | 0.645-0.962 | 0.727 | 0.893 |
| DBIL | 23.50 | 0.829 | 0.691-0.968 | 0.727 | 0.912 |
| AST | 119.30 | 0.687 | 0.484-0.889 | 0.545 | 0.921 |
| WBC | 18.03 | 0.708 | 0.520-0.897 | 0.545 | 0.899 |
| NE% | 85.28 | 0.773 | 0.632-0.914 | 0.909 | 0.625 |
| HGB | 102.50 | 0.827 | 0.726-0.928 | 0.909 | 0.748 |
| PLT | 67.0 | 0.737 | 0.548-0.927 | 0.636 | 0.931 |
| PT | 17.65 | 0.813 | 0.641-0.984 | 0.636 | 0.968 |
| APTT | 33.15 | 0.863 | 0.727-1.000 | 0.909 | 0.760 |
| FIB | 3.50 | 0.774 | 0.627-0.921 | 0.545 | 0.909 |

Abbreviations: TC, total cholesterol; HDL-C, high-density lipoprotein cholesterol; LDL-C, low-density lipoprotein cholesterol; TG, triglyceride; Lp(a), lipoprotein(a); TBIL, total bilirubin; DBIL, direct bilirubin; AST, aspartate transaminase; WBC, white blood cell count; NE%, neutrophil percentage; HGB, hemoglobin; PLT, platelet; PT, prothrombin time; APTT, activated partial thromboplastin time; FIB, fibrinogen.


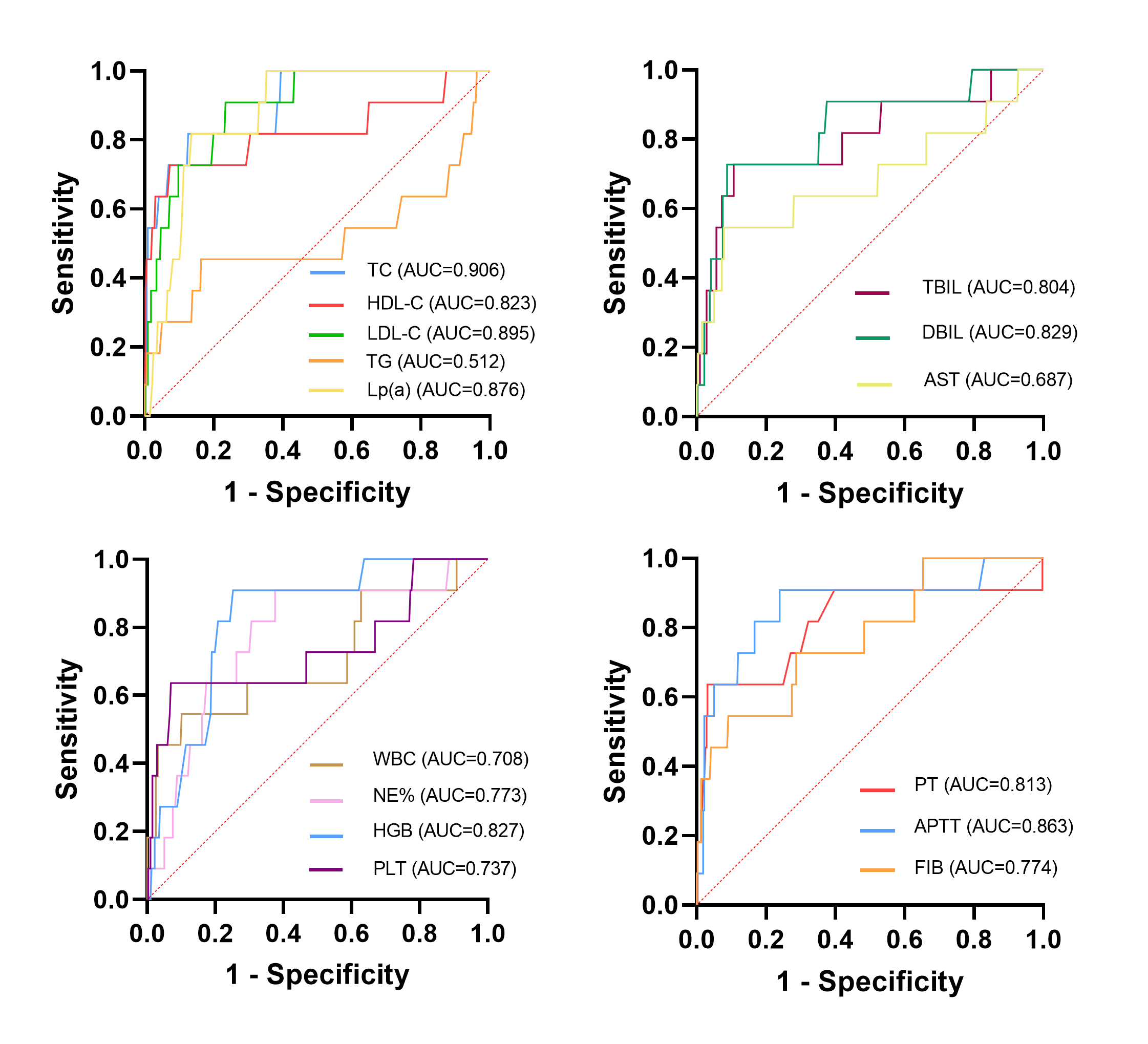


Figure S1. Receiver operating characteristic (ROC) curves for the prediction of death by laboratory indicators routinely measured in patients with PLA.

Abbreviations: TC, total cholesterol; HDL-C, high-density lipoprotein cholesterol; LDL-C, low-density lipoprotein cholesterol; TG, triglyceride; Lp(a), lipoprotein(a); TBIL, total bilirubin; DBIL, direct bilirubin; AST, aspartate transaminase; WBC, white blood cell count; NE%, neutrophil percentage; HGB, hemoglobin; PLT, platelet; PT, prothrombin time; APTT, activated partial thromboplastin time; FIB, fibrinogen.
